# Impact of automatic acquisition of key clinical information on the accuracy of electrocardiogram interpretation: a cross-sectional study

**DOI:** 10.1186/s12909-023-04907-9

**Published:** 2023-12-08

**Authors:** Shaohua Guo, Bufan Zhang, Yuanyuan Feng, Yajie Wang, Gary Tse, Tong Liu, Kang-Yin Chen

**Affiliations:** 1 Tianjin Key Laboratory of Ionic-Molecular Function of Cardiovascular disease, Department of Cardiology, Tianjin Institute of Cardiology, The Second Hospital of Tianjin Medical University, 23, Pingjiang Road, Hexi District, Tianjin, 300211 People’s Republic of China; 2 Department of Cardiovascular Surgery, Tianjin Medical University General Hospital, Tianjin, China; 3 Department of Cardiology, TEDA International Cardiovascular Hospital, Cardiovascular Clinical College of Tianjin Medical University, Tianjin, People’s Republic of China; 4 Cardiac Electrophysiology Unit, Cardiovascular Analytics Group, China-UK Collaboration, Hong Kong, China; 5 Kent and Medway Medical School, Canterbury, UK; 6 School of Nursing and Health Studies, Metropolitan University, Hong Kong, China; 7 The School of Precision Instrument and Opto-electronic Engineering, Tianjin University, Tianjin, 300072 China

**Keywords:** Artificial intelligence, Electrocardiogram interpretation, Key clinical information

## Abstract

**Background:**

The accuracy of electrocardiogram (ECG) interpretation by doctors are affected by the available clinical information. However, having a complete set of clinical details before making a diagnosis is very difficult in the clinical setting especially in the early stages of the admission process. Therefore, we developed an artificial intelligence-assisted ECG diagnostic system (AI-ECG) using natural language processing to provide screened key clinical information during ECG interpretation.

**Methods:**

Doctors with varying levels of training were asked to make diagnoses from 50 ECGs using a common ECG diagnosis system that does not contain clinical information. After a two-week-blanking period, the same set of ECGs was reinterpreted by the same doctors with AI-ECG containing clinical information. Two cardiologists independently provided diagnostic criteria for 50 ECGs, and discrepancies were resolved by consensus or, if necessary, by a third cardiologist. The accuracy of ECG interpretation was assessed, with each response scored as correct/partially correct = 1 or incorrect = 0.

**Results:**

The mean accuracy of ECG interpretation was 30.2% and 36.2% with the common ECG system and AI-ECG system, respectively. Compared to the unaided ECG system, the accuracy of interpretation was significantly improved with the AI-ECG system (P for paired t-test = 0.002). For senior doctors, no improvement was found in ECG interpretation accuracy, while an AI-ECG system was associated with 27% higher mean scores (24.3 ± 9.4% vs. 30.9 ± 10.6%, P = 0.005) for junior doctors.

**Conclusion:**

Intelligently screened key clinical information could improve the accuracy of ECG interpretation by doctors, especially for junior doctors.

**Supplementary Information:**

The online version contains supplementary material available at 10.1186/s12909-023-04907-9.

## Introduction

Electrocardiogram (ECG) interpretation is a vital component of clinical medicine and its accurate interpretation is important for maintaining high standards of patient care. Errors in ECG interpretation can lead to severe consequences, such as delays in the revascularization of occluded coronary arteries in patients with acute myocardial infarction and the reconigition of the significant long QT interval. However, there is compelling evidence that doctors have significant error rates in ECG diagnosis. According to a meta-analysis, the accuracy of ECG interpretation by doctors or medical students varied widely across all training levels (4-95%). The median accuracy was 42% for medical students and 74.9% for cardiologists. [1] Even cardiologists cannot be deemed fully competent in electrocardiographic interpretation. Viskin et al. [2] investigated doctors’ capability of distinguishing long QT interval, a life-threatening condition, finding that most doctors, including many cardiologists, cannot correctly identify a long QT. Another study reported the overall diagnostic accuracy was only 58% for cardiology residents [3].

Some studies of ECG interpretation have shown that providing a clinical history may impact doctors’ diagnostic accuracy. In one study, three cardiologists wrote an interpretation of a set of 52 ECGs devoid of clinical information and three weeks later interpreted the same ECGs with a clinical history [4]. As a result, 14% of the initial ECGs were interpreted with a different diagnosis. Hatala et al. explored the effect of clinical information on doctors’ ECG interpretation skills in 1996 [5] and 1999 [6]. The results showed that for doctors with different levels of expertise, a correct history could improve the accuracy of ECG interpretation, while providing a misleading history could also reduce accuracy, especially for junior doctors. Notwithstanding that a prior study have also demonstrated a weak influence of clinical information on the interpretation of ECGs [5], the most recent study with a large sample supported the positive influence of clinical history on the accuracy of ECG interpretation [6].

In real-world clinical practice, ECGs are rarely interpreted without knowledge of the clinical background of the cases. There are features that may leave subtle effects on the ECG waveforms or enable these to be detected, such as the availability of prior ECGs, laboratory assays, common comorbidities, medication, and echocardiography. Unfortunately, it is impossible to comprehensively grasp every detail in the entire record before interpreting an ECG. Therefore, we developed an artificial intelligence-assisted ECG diagnostic system (AI-ECG) using natural language processing to extract key and concise clinical information for doctors during ECG interpretation.

This study aimed to evaluate whether AI-ECG with automatically acquired key clinical information can assist doctors in improving the accuracy of ECG interpretation compared with the common ECG interpretation system.

## Methods

### Participants

We performed a cross-sectional study on postgraduate trainees (including first-, second-, and third-year postgraduate training) and cardiologists from the Second Hospital of Tianjin Medical University. Participants voluntarily performed the tests.

### Testing ECG

An ECG test package containing 50 ECGs was created. The package covers a core syllabus of cardiovascular diagnoses, which include arrhythmias (blocked premature ventricular contractions, premature atrial contractions, first-degree atrioventricular block, Mobitz type I and II second-degree atrioventricular block, complete atrioventricular block, High-degree atrioventricular block, atrial tachycardia, atrial fibrillation, atrial flutter, atrioventricular reentrant tachycardia, ventricular tachycardia, accelerated idioventricular rhythm and Torsade de pointes), and other disease which can cause waveform abnormalities (left atrial enlargement caused by mitral stenosis, left ventricular hypertrophy caused by hypertension, aortic stenosis and hypertrophic cardiomyopathy, right ventricular hypertrophy caused by pulmonary hypertension, S_I_Q_III_T_III_ caused by pulmonary embolism, left bundle branch block, right bundle branch block, AF with Wolff-Parkinson-White pattern, ST-segment elevation myocardial infarction, hypokalaemia, long QT syndrome caused by amiodarone, Brugada syndrome, De Winter syndrome, pacing rhythm, Epsilon-waves caused by arrhythmogenic right ventricular cardiomyopathy, and atypical waveform abnormalities caused by non ST-segment elevation myocardial infarction and heart failure). The details of this ECG test package are listed in Supplementary Table 1.

### Artificial intelligence-assisted ECG diagnostic system and common ECG diagnostic system

**Fig. 1:**
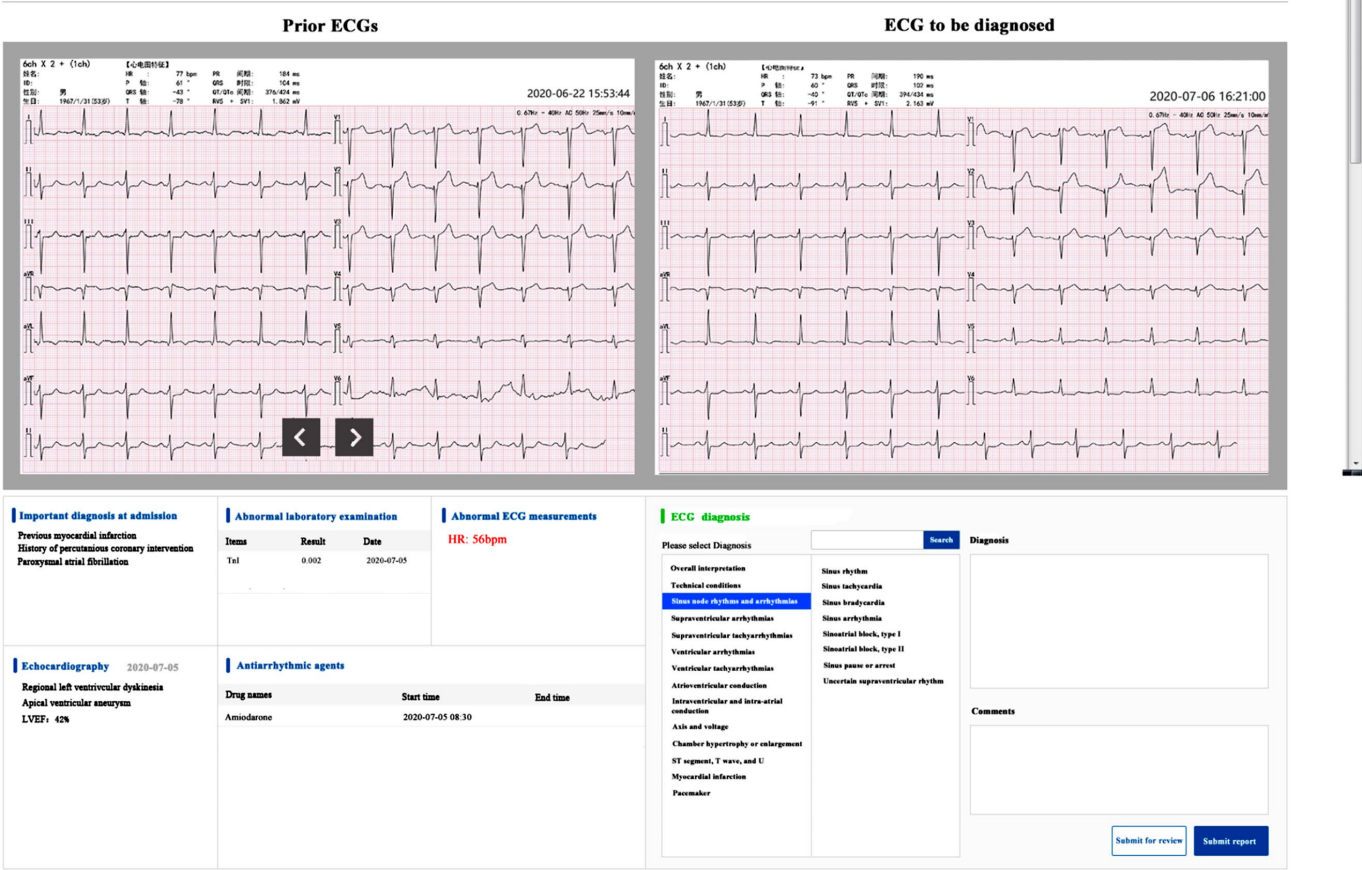
The interface of artificial intelligence-assisted ECG diagnostic system. ECG, electrocardiograph

The AI-ECG diagnostic interface is divided into four quadrants (Fig. 1). The upper left quadrant displays the prior ECGs, which can be paged if multiple prior ECGs exist. The upper right quadrant shows the ECG to be diagnosed this time; the lower left quadrant shows the patient’s key information tags, which are screened by an AI model. Based on theoretical medical knowledge as well as clinical experience, we trained the AI model using machine learning to filter out clinical key information that may leave a trace in the ECG, including preliminary cardiovascular diagnosis, laboratory tests, cardiac ultrasound diagnosis, abnormal ECG measurements, and clinical medications. The contents of the lower left quadrant tags are subdivided into five sections of related diagnoses, abnormal cardiac echocardiographic indications, related abnormal laboratory indications, related abnormal ECG values, and related medication indications. The rules for AI learning and extracting tags are set in advance. The lower right quadrant is a template of ECG diagnostic terms based on the AHA consensus [7, 8], which allows doctors to select the appropriate diagnosis and leave comments.

The interface of a common ECG diagnostic system is similar to AI-ECG system but without the tabs in the lower left quadrant.

### Study design

During the timeframe between June 2021 and December 2021, the AI-ECG system was meticulously developed and underwent extensive testing. In the subsequent period from January 2022 to March 2022, a specific ECG testing dataset was carefully selected. Finally, from April 2022 to June 2022, doctors were recruited for clinical study.

**Fig. 2:**
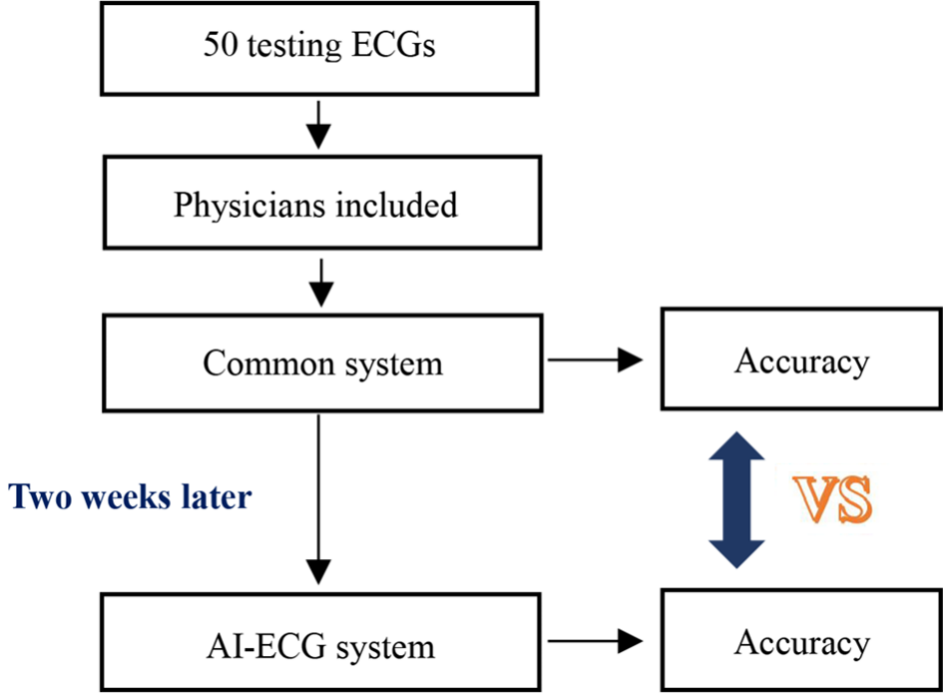
Study flow chart. ECG, electrocardiograph; AI-ECG system, artificial intelligence-assisted ECG diagnostic system

The clinical study flow is outlined in Fig. 2. Doctors were asked to independently interpret the set of 50 ECGs using a common ECG diagnosis system devoid of any clinical information. They were not told the purpose of the study. After a two-week washout period, the same set of ECGs was reinterpreted by each physician using the AI-ECG platform. The doctors were unaware that the same set of ECGs was used. Two cardiologists independently gave diagnostic criteria for 50 ECGs, and discrepancies were resolved by consensus or, if necessary, by a third cardiologist. Each response was scored by one of the investigators according to the criteria. As most ECGs have more than one answer, diagnostic accuracy was graded as correct in all answers/ in main answers = 1 or incorrect = 0. If multiple diagnoses including the correct main answers were listed, the item was scored as correct.

### Statistical analysis

The study was designed as a matched pair study. SPSS statistical software (SPSS 25.0, SPSS Inc., Chicago, IL, USA) and R programming (version 4.2.2; R Foundation for Statistical Computing, Vienna, Austria) were used for the statistical analysis. P-values of 0.05 or lower were considered statistically significant. Unmodified Kolmogorov-Smirnov goodness-of-fit tests were used to test for the distribution of the variables. Since each ECG was interpreted twice by the common ECG diagnosis system and AI-ECG platform respectively, the analysis had to be perceived as a matched pair design, which led to the student paired t-test or non-parametric Wilcoxon rank-sum tests for the method comparison.

## Results

**Fig. 3:**
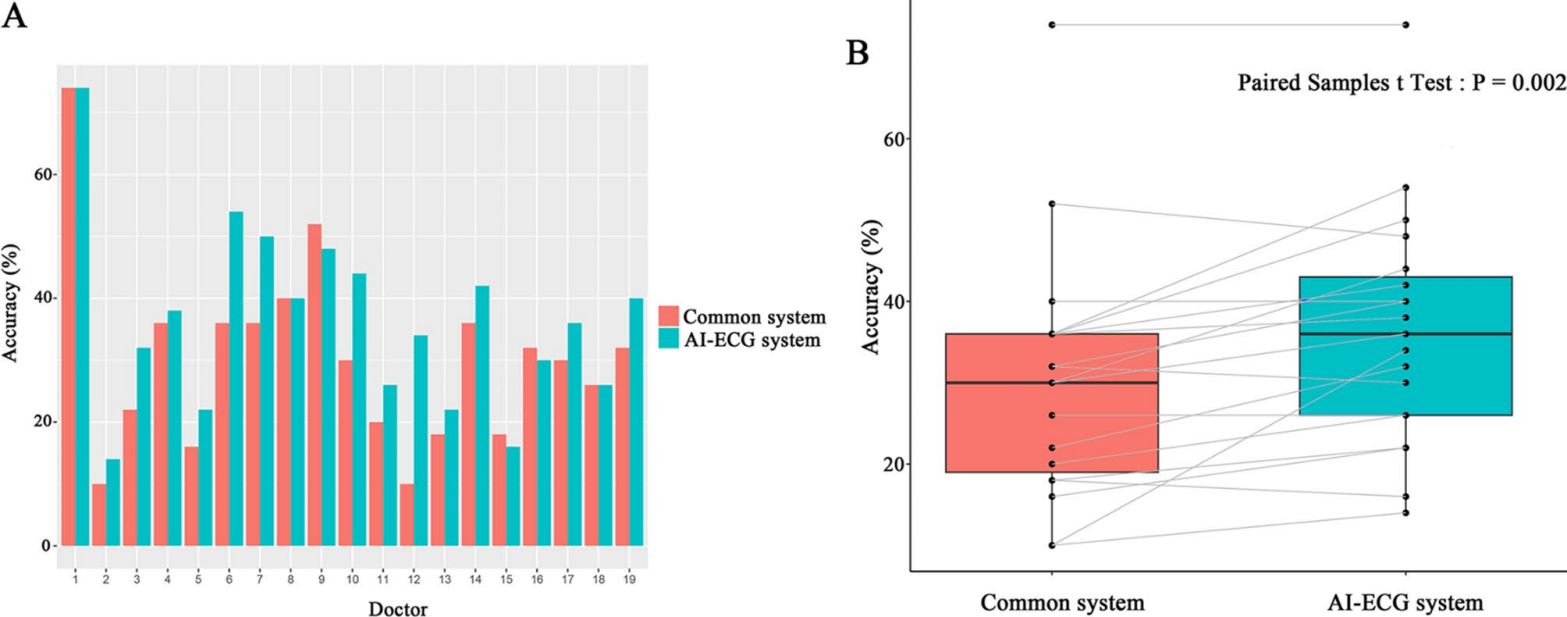
Diagnostic accuracy in all included doctors. (**A**) a bar chart showing individual accuracy of ECG interpretation by the common system and AI-ECG system; (**B**) a boxplot graph showing the accuracy of the common system compared to the accuracy of the AI-ECG system

A total of 19 doctors were included in this study, of whom five were cardiologists and 14 were postgraduate trainees, representing all 3 training years (4 first-, 3 s-, and 7 third-year postgraduate training). ECG interpretation accuracy for testing ECGs of each doctor is shown in Table 1. The mean diagnostic accuracy for all 50 diagnoses was 30.21% by the common ECG diagnosis system and 36.21% by the AI-ECG platform. As shown in Fig. 3, the overall accuracy using the AI-ECG platform with the help of key clinical information was significantly improved compared to the common ECG diagnosis system (student paired t-test, 36.2 ± 14.5% vs. 30.2 ± 15.2%, P = 0.002).

**Table 1:** The ECG interpretation accuracy of each physicians

		Diagnostic Classification													
Physicians	Senior or junior	All ECGs		Life threatening diagnosis		Arrhythmia		Hypertrophy		Ischemia, or infarction		Conduction disturbances		others	
		Common-ECG	AI-ECG	Common-ECG	AI-ECG	Common-ECG	AI-ECG	Common-ECG	AI-ECG	Common-ECG	AI-ECG	Common-ECG	AI-ECG	Common-ECG	AI-ECG
01	cardiologists	37 (74%)	37 (74%)	8 (80%)	8 (80%)	15 (83.33%)	16 (88.89%)	2 (40%)	1 (20%)	10 (71.43%)	11 (78.57%)	7 (100%)	6 (85.71%)	3 (50%)	3 (50%)
02	trainees	5 (10%)	7 (14%)	3 (30%)	3 (30%)	2 (11.11%)	3 (16.67%)	0 (0%)	0 (0%)	2 (14.29%)	3 (21.43%)	1 (14.29%)	2 (28.57%)	1 (16.67%)	0 (0%)
03	trainees	11 (22%)	16 (32%)	4 (40%)	6 (60%)	7 (38.89%)	5 (27.78%)	0 (0%)	1 (20%)	2 (14.29%)	5 (35.71%)	2 (28.57%)	4 (57.14%)	0 (0%)	1 (16.67%)
04	cardiologists	18 (36%)	19 (38%)	7 (70%)	5 (50%)	7 (38.89%)	8 (44.44%)	1 (20%)	0 (0%)	3 (21.43%)	4 (28.57%)	5 (71.43%)	4 (57.14%)	1 (16.67%)	0 (0%)
05	trainees	8 (16%)	11 (22%)	3 (30%)	2 (20%)	5 (27.78%)	4 (22.22%)	0 (0%)	1 (20%)	1 (7.14%)	6 (42.86%)	2 (28.57%)	0 (0%)	0 (0%)	0 (0%)
06	cardiologists	18 (36%)	27 (54%)	4 (40%)	6 (60%)	10 (55.56%	12 (66.67%)	0 (0%)	3 (60%)	4 (28.57%)	4 (28.57%)	2 (28.57%)	6 (85.71%)	1 (16.67%)	2 (33.33%)
07	trainees	18 (36%)	25 (50%)	3 (30%)	4 (40%)	8 (44.44%)	12 (66.67%)	2 (40%)	2 (20%)	4 (28.57%)	5 (35.71%)	2 (28.57%)	2 (28.57%)	2 (33.33%)	4 (66.67%)
08	trainees	20 (40%)	20 (40%)	7 (70%)	7 (70%)	8 (44.44%)	6 (33.33%)	1 (20%)	0 (0%)	4 (28.57%)	8 (57.14%)	6 (85.71%)	6 (85.71%)	1 (16.67%)	0 (0%)
09	cardiologists	26 (52%)	24 (48%)	7 (70%)	6 (60%)	11 (61.11%	9 (50%)	1 (20%)	3 (60%)	8 (57.14%)	3 (21.43%)	5 (71.43%)	6 (85.71%)	1 (16.67%)	3 (50%)
10	trainees	15 (30%)	22 (44%)	5 (50%)	5 (50%)	8 (44.44%)	8 (44.44%)	1 (20%)	2 (40%)	1 (7.14%)	5 (35.71%)	4 (57.14%)	4 (57.14%)	1 (16.67%)	2 (33.33%)
11	trainees	10 (20%)	13 (26%)	3 (30%)	2 (20%)	5 (27.78%)	6 (33.33%)	0 (0%)	0 (0%)	2 (14.29%)	4 (28.57%)	3 (42.86%)	2 (28.57%)	0 (0%)	1 (16.67%)
12	trainees	5 (10%)	17 (34%)	2 (20%)	7 (70%)	4 (22.22%)	6 (33.33%)	0 (0%)	1 (20%)	0 (0%)	4 (28.57%)	1 (14.29%)	5 (71.43%)	0 (0%)	1 (16.67%)
13	trainees	9 (18%)	11 (22%)	2 (20%)	2 (20%)	4 (22.22%)	7 (38.89%)	0 (0%)	0 (0%)	2 (14.29%)	1 (7.14%)	2 (28.57%)	3 (42.86%)	0 (0%)	1 (16.67%)
14	cardiologists	18 (36%)	21 (42%)	3 (30%)	5 (50%)	7 (38.89%)	9 (50%)	1 (20%)	0 (0%)	3 (21.43%)	5 (35.71%)	6 (85.71%)	6 (85.71%)	0 (0%)	1 (16.67%)
15	trainees	9 (18%)	8 (16%)	3 (30%)	3 (30%)	2 (11.11%)	4 (22.22%)	0 (0%)	0 (0%)	3 (21.43%)	1 (7.14%)	4 (57.14%)	2 (28.57%)	0 (0%)	0 (0%)
16	trainees	16 (32%)	15 (30%)	3 (30%)	3 (30%)	5 (27.78%)	6 (33.33%)	2 (40%)	0 (0%)	3 (21.43%)	3 (21.43%)	6 (85.71%)	6 (85.71%)	0 (0%)	0 (0%)
17	trainees	15 (30%)	18 (36%)	3 (30%)	4 (40%)	7 (38.89%)	7 (38.89%)	2 (40%)	3 (60%)	0 (0%)	2 (14.29%)	6 (85.71%)	5 (71.43%)	0 (0%)	0 (0%)
18	trainees	13 (26%)	13 (36%)	5 (50%)	4 (40%)	7 (38.89%)	7 (38.89%)	0 (0%)	1 (20%)	1 (7.14%)	1 (7.14%)	5 (71.43%)	4 (57.14%)	0 (0%)	0 (0%)
19	trainees	16 (32%)	20 (40%)	3 (30%)	6 (60%)	8 (44.44%)	9 (50%)	1 (20%)	1 (20%)	3 (21.43%)	6 (42.86%)	4 (57.14%)	4 (57.14%)	0 (0%)	0 (0%)

ECG, Electrocardiograph; AI-ECG, artificial intelligence-assisted ECG diagnostic system

Of 10 life-threatening diagnoses including Brugada syndrome, complete atrioventricular block, long QT interval, atrial fibrillation combined with Wolff–Parkinson–White syndrome, Torsades de Pointes, and ventricular tachycardia, the mean accuracy was only improved by 5.26% using the AI-ECG system compared to the common system without statistical significance (P = 0.188).

**Fig. 4:**
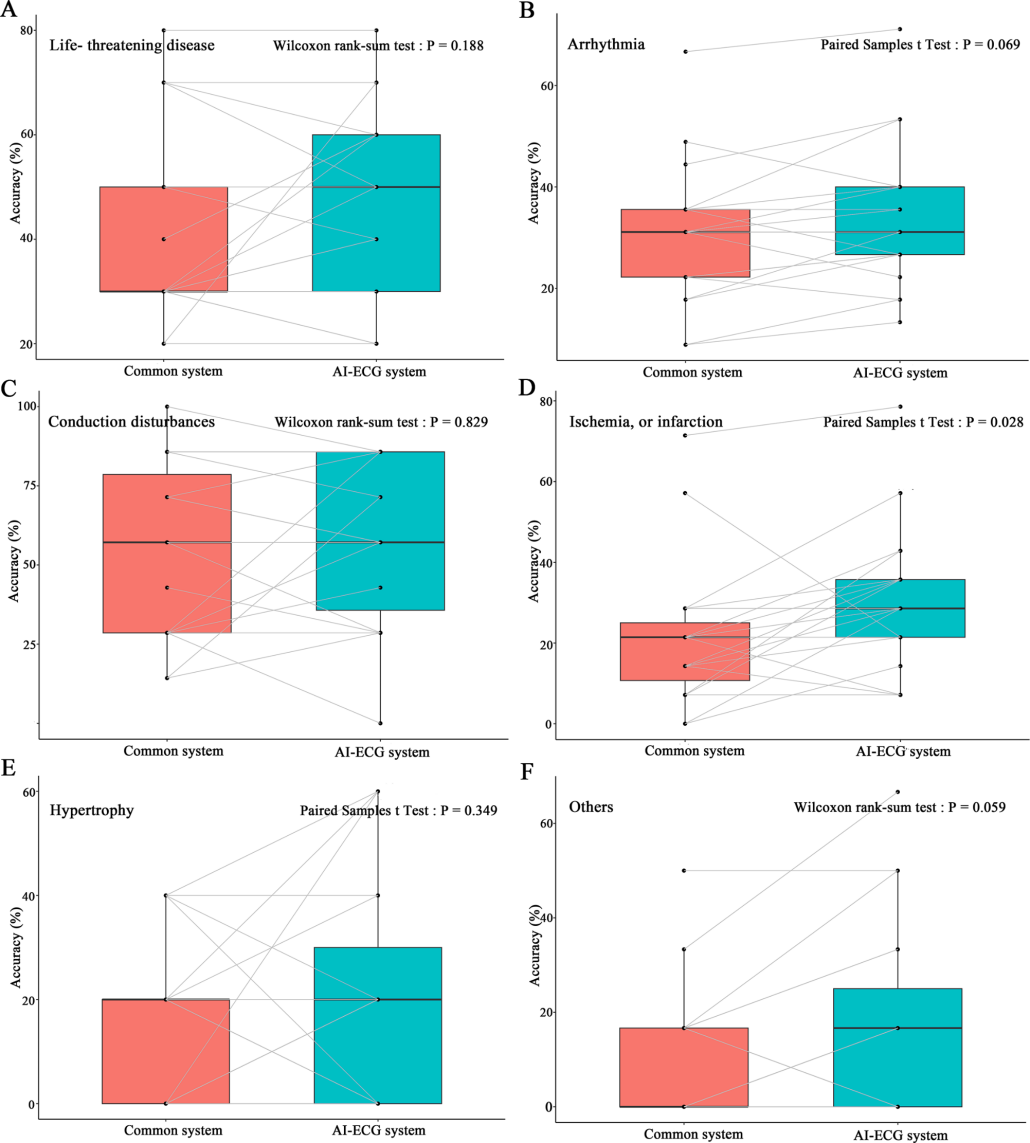
Comparison between common system and AI-ECG system on accuracy of ECG interpretation in various diagnostic categories. (**A**) life-threatening disease; (**B**) arrhythmia; (**C**) conduction disturbances; (**D**) ischemia or infarction; (**E**) hypertrophy; (**F**) others

Testing ECGs were divided into five categories based on the main diagnosis: (1) arrhythmia; (2) conduction disturbances; (3) hypertrophy; (4) ischemia or infarction; and (5) others (including Brugada syndrome, S1Q3T3 pattern, Epsilon wave, long QT interval, and Takotsubo cardiomyopathy). The AI-ECG system can significantly improve the diagnostic accuracy of ECGs about ischemia or infarction (P = 0.028) and has a trend toward improving the diagnostic accuracy of arrhythmia (P = 0.069) and other special conditions (P = 0.059), while this phenomenon was not prominent in the categories of conduction disturbances, and hypertrophy (Fig. 4).

**Fig. 5:**
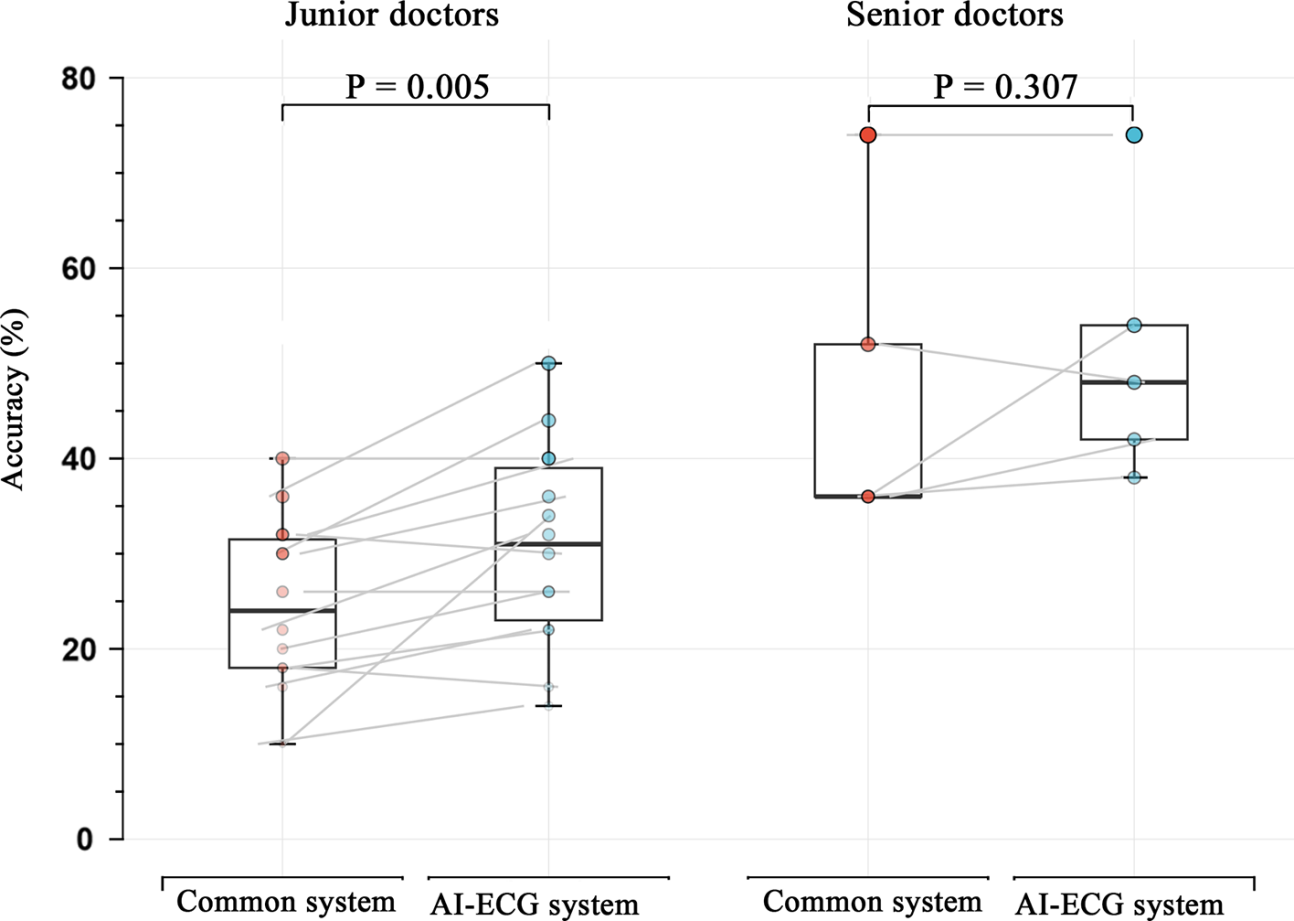
Subgroup analysis by cardiology training. Junior doctors were postgraduate trainees and senior doctors were cardiologists

The overall diagnostic accuracy of interpretation differed depending on the level of cardiology training. Subgroup analysis shows that the postgraduate trainees’ accuracy in ECG interpretation was significantly improved using the AI-ECG system (24.3 ± 9.4% vs. 30.9 ± 10.6%, P = 0.005). However, no difference in ECG interpretation accuracy of cardiologists was observed either using the common system or AI-ECG system (46.8 ± 16.7% vs. 51.2 ± 14.1%, P = 0.307) (Fig. 5). Surprisingly, cardiologists are more likely to change the correct answer to the wrong one with more clinical information using the AI-ECG system (P = 0.009) (Table 2).

**Table 2:** Number of ECGs reinterpreted with a different diagnosis for the second time

	Cardiologists	Postgraduate trainees	P-value
The number of increased correct answers	9.4 ± 3.9	6.9 ± 3.4	0.184
The number of increased wrong answers	7.2 ± 2.8	4.0 ± 1.8	0.009

## Discussion

Compared to the common ECG diagnostic system, we have demonstrated a strong and advantageous effect of artificial intelligence-assisted ECG diagnostic system on the accuracy of ECG interpretation. To our knowledge, this is the first bold attempt to explore a new way of changing and improving the diagnosis based on the original ECG, providing a proof-of-concept of introducing artificial intelligence for real-world applications in the clinical environment.

The AI-ECG system can significantly improve the accuracy of doctors’ ECG diagnoses comparing to the common system, which can be attributed to the following reasons: (1) The system integrates the clinical expertise and wisdom of experts when extracting key clinical information; (2) It significantly reduces the time doctors spend on reviewing complex clinical information, thereby improving efficiency and minimizing omissions; (3) The information is well synchronized in AI-ECG system, as the latest clinical information is extracted based on the completion time of the electrocardiogram. It should be noted that although the AI-ECG system accurately extracts the key clinical information required for ECG diagnosis, the accuracy of the diagnosis primarily relies on the level of training and expertise of the doctor. Compared to common systems, this AI-ECG system, when used for training doctors, enables the integration of ECG and clinical knowledge, efficient error correction, and potentially the development of more personalized training modes in the future.

As summarized by a systematic review and meta-analysis in 2020, which identified 78 studies on the accuracy of ECG interpretations by doctors, accuracy scores varied widely across studies, ranging from 4 to 95%, and the median accuracy across all training levels was relatively low (54%) and scores increased as expected with progressive training and specialization [1]. Our study reported 30.2% mean ECG diagnostic accuracy. Even with the help of the AI-ECG system, the mean accuracy only increased to 36.2%. The performance of doctors in this study seems not to be as good as in previous studies. However, the ECGs included in this study were much more complicated than those in previous studies, which can be explained by the following aspects. First, a larger number of testing ECGs than before [9–11], and 28% had more than one main diagnosis, which meant that the disease was more complicated to diagnose. Furthermore, the ECG came from actual inpatients in the department of cardiology, leading to atypical ECGs affected by treatment and other conditions. Third, most doctors (58%) have not yet completed 3 years of postgraduate internal medicine training. Fourth, although participants volunteered to participate in the study, it is important to consider the variations in their mindset and determination to complete the study as they face the challenges of diagnosing a large number of complex ECG.

Although previous reports have suggested that there is only a minimal clinical effect of ECG misinterpretation [12], the wrong diagnosis may potentially expose patients to unnecessary additional testing, incorrect or delayed treatment, and overlook potentially life-threatening conditions of long QT interval and ventricular tachycardia. Of note, this study’s subgroup analysis indicates that AI-assisted ECG diagnostic systems did not improve the accuracy of life-threatening ECG diagnosis. The importance of being able to recognize a life-threatening disease using ECG cannot be overemphasized. Though the limited number of participating doctors may have an impact, the improvement of AI-ECG and the specialization of doctors are also important.

Otherwise, though the performance of cardiologists was significantly better than postgraduate trainees in ECG interpretation with complex ECGs, the accuracy rate of 51.2% in this study remains unsatisfactory. As far back as 2005^2^, studies cast doubt on doctors’ ability to interpret ECGs, and Sibbald et al. in 2014^3^ further suggested that cardiologists may not be as competent in ECG interpretation as we thought. Therefore, AI-assisted ECG diagnostic systems need to be further improved, not only to identify and extract key clinical information such as critical QT prolongation but also to design more effective reminders to ensure that doctors are fully aware of this finding. In addition, it is imperative to explore the value of AI automatic diagnosis systems in medical student and resident training, providing doctors with a more intelligent and efficient training platform.

## Limitations

Firstly, only 19 doctors were studied, and the performance of the AI-ECG system could have been better explored if more doctors with different cardiology training had been included. Moreover, the real-world clinical environment is often more complex. Postgraduate trainees with limited cardiology training may have trouble processing the abnormal information extracted by AI. Finally, this was not a critical examination, increasing the possibility that an individual’s motivation to complete the examination to the best of their ability might have been varied.

## Conclusion

Our AI-ECG system, which intelligently extracts and summarizes key clinical information that may leave a trace in ECG, can significantly improve the accuracy of ECG interpretation by doctors. There was a significant effect of expertise on diagnostic accuracy but no effect of the AI-ECG system on cardiologists, suggesting that efforts should be made at all levels of medical education to increase the awareness and knowledge of the medical community about ECG interpretation.

### Electronic supplementary material

Below is the link to the electronic supplementary material.

**Supplementary Material 1: **Main diagnosis and AI screened key information of testing electrocardiogram

## Data Availability

The datasets used during the current study are available from the corresponding author on reasonable request.

## List of abbreviations

ECG: electrocardiogram
AI-ECG: artificial intelligence-assisted ECG diagnostic system
