# Supplementary material for: Impact of automatic acquisition of key clinical information on the accuracy of electrocardiogram interpretation: a cross-sectional study
Source: BMC Med Educ. 2023 Dec 8;23:936. doi: 10.1186/s12909-023-04907-9 (PMC10709941; doi:10.1186/s12909-023-04907-9)
Supplement: Supplementary file 1 — Supplementary Material 1: Main diagnosis and AI screened key information of testing electrocardiogram [file 12909_2023_4907_MOESM1_ESM.docx]

Main diagnosis of testing electrocardiograph

| Diagnostic Group | Testing ECG Number | Main diagnosis | | AI screened key information | | | | |
| --- | --- | --- | --- | --- | --- | --- | --- | --- |
|  |  |  |  | Diagnosis at admission | Abnormal ECG measurements | Abnormal laboratory examination and echocardiography | Antiarrhythmic agents | history elecatrocargiogram |
| Arrhythmia | 1 | Atrial fibrillation | Complete left bundle branch block | atrial fibrillation | HR:138bpm;  QRS:140ms | Left atrial enlargement |  |  |
|  | 2 | Multiform premature ventricular contraction |  | History of ablation for supraventricular Tachycardia |  | Left atrial enlargement | amiodarone | 5 days ago |
|  | 3 | Atrial pacing |  | History of pacemaker implantation | QT:464ms |  |  | 1 day ago |
|  | 4 | Blocked premature atrial contractions |  | Bradycardia | HR:53bpm | HR: 53bpm |  |  |
|  | 5 | Atrial tachycardia |  | Supraventricular Tachycardia | HR：122bpm  PR：145ms | Right and left atrial enlargement |  |  |
|  | 6 | Interpolated premature ventricular contraction | premature atrial contraction | Paroxysmal atrial fibrillation | PR:234ms  QT：470ms | Left atrial enlargement  Left ventricular hypertrophy |  | 2 days ago |
|  | 7 | Accelerated idioventricular rhythm |  |  |  | cTnI:2.683ng/ml |  |  |
|  | 8 | Atrial fibrillation | Previous anteroseptal wall myocardial infarction | History of percutanious coronary intervention and pacemaker implantation | HR:128bpm  QT:478ms | LVEF:40%  ventricular aneurysm; | amiodarone | 7days ago |
|  | 9 | Atrial fibrillation | Wolff–Parkinson–White syndrome | Supraventricular Tachycardia | HR:166bpm  PR:177ms  QT:520ms | TnI:0.061ng/ml |  | 30 minutes ago |
|  | 10 | Torsades de Pointes |  | Atrial fibrillation | HR:120bpm  QT: 791ms |  | amiodarone |  |
|  | 11 | Orthodromic atrioventricular reentrant tachycardia |  | Supraventricular Tachycardia | HR: 220bpm |  |  | 10 minutes ago |
|  | 12 | Multiform ventricular tachycardia |  | Heart failure | HR:156bpm | LVEF: 19% | cedilanid |  |
|  | 13 | Ventricular tachycardia |  | Previous myocardial infarction | HR：180bpm | Regional left ventrivcular dyskinesia |  |  |
|  | 14 | Atrial fibrillation | Premature ventricular contraction | Heart failure | PR:228ms | LVEF:21%  Heart enlargement |  | 2 days ago |
|  | 15 | Atrial fibrillation | Premature ventricular contraction | Atrial fibrillation |  | Left atrial enlargement | metoprolol | 5 days go |
|  | 16 | Atrial flutter |  | Tricuspid valvular insufficiency | HR:54bpm | Mitral valve replacement | bisoprolol | 6 days ago |
|  | 17 | Atrial sensing, ventricular pacing |  | History of pacemaker implantation  Complete left bundle branch block |  |  |  | 3 months and 6 months ago |
|  | 18 | Atrial pacing, ventricular sensing |  | History of pacemaker implantation  Dilated cardiomyopathy | QRS:150ms  QT:462ms | LVEF: 39%;  Left heart enlargement | esmolol |  |
| Conduction disturbances | 19 | Complete atrioventricular block |  | Hypertension | HR:37 bpm  QT: 640ms | K:3.4mmol/L |  |  |
|  | 20 | High-degree atrioventricular block |  | Syncope | HR: 30bpm  QT: 520/367ms |  |  |  |
|  | 21 | Complete atrioventricular block | Atrial fibrillation | Atrial fibrillation | HR: 34bpm  QT: 600/451ms | Left atrial enlargement | digoxin  metoprolol |  |
|  | 22 | First-degree atrioventricular block |  | Coronary artery disease | PR:226ms  QT: 488ms |  | Ssotalol |  |
|  | 23 | Mobitz type II second-degree atrioventricular block |  | Complete right bundle branch block | QRS:140ms | Calcification of aortic valves  Moderate aortic regurgitation |  |  |
|  | 24 | Complete left bundle branch block |  | Hypertension | QT:450ms  QRS:168ms | Left ventricular hypertrophy |  | 3 days ago |
|  | 25 | Complete right bundle branch block |  | Previous myocardial infarction | PR:302ms  QT:484ms  QRS:174ms |  |  | 1 day ago |
| Ischemia, or infarction | 26 | De Winter syndrome |  | Acute coronary syndrome |  | TnI:1.26ng/ml  Regional left ventrivcular dyskinesia |  |  |
|  | 27 | Acute inferior wall myocardial infarction | Mobitz type I second-degree atrioventricular block | Acute coronary syndrome | QT: 408/470ms | TnI: 2.55ng/ml  Regional left ventrivcular dyskinesia |  |  |
|  | 28 | Wellens syndrome |  | Unstable angina | QT: 440/556ms |  |  |  |
|  | 29 | Acute inferior wall myocardial infarction | Complete atrioventricular block | Acute myocardial infarction | HR:37bpm  QT: 480ms | TnI:2.786ng/ml;  Regional left ventrivcular akinesia |  |  |
|  | 30 | Acute inferior wall myocardial infarction |  | Acute myocardial infarction |  | TnI: 2.55ng/ml |  |  |
|  | 31 | Acute anterior wall myocardial infarction | Acute inferior wall myocardial infarction | Acute myocardial infarction |  | LVEF: 39%  Regional left ventrivcular dyskinesia;  Apical ventricular aneurysm |  | 2, 4, 6 and 9 days ago |
|  | 32 | Acute inferior wall myocardial infarction |  | Acute inferior wall myocardial infarction |  | TnI:0.59ng/ml  Regional left ventrivcular akinesia; |  | 1, 2, 3, 4, and 5 days ago |
|  | 33 | ST-T segment alterations |  | Unstable angina pectoris |  | TnI:3.795ng/ml |  | 2 days ago |
|  | 34 | ST-T segment alterations | Multiform premature ventricular contraction | Acute coronary syndrome |  | TnI: 7.193ng/ml  Regional left ventrivcular akinesia; |  | 2 days ago |
|  | 35 | Previous anterior wall myocardial infarction |  | Previous myocardial infarction  hypertension |  | Regional left ventrivcular akinesia; |  | 1 day ago |
|  | 36 | Previous anteroseptal wall myocardial infarction | ST-T segment alterations | Previous myocardial infarction  History of percutanious coronary intervention  Paroxysmal atrial fibrillation | PR：202ms  QT: 516ms | Apical ventricular aneurysm |  | 5 days ago |
|  | 37 | Previous anteroseptal wall myocardial infarction | ST-T segment alterations | Unstable angina pectoris | HR: 53bpm  QT: 576ms | Regional left ventrivcular akinesia | bisoprolol | 20 days ago |
|  | 38 | Ventricular aneurysm |  | Previous myocardial infarction  Hypertension  Ventricular aneurysm | PR: 212ms  QT: 514ms | LVEF: 34%  Apical ventricular aneurysm; |  | 4 days ago |
|  |  |  |  |  |  |  |  |  |
| Hypertrophy | 39 | Previous anteroseptal wall myocardial infarction |  | Previous myocardial infarction  Heart failure |  | LVEF: 43%;  Left heart enlargement |  | 9 and 15 days ago |
|  | 40 | Left atrial enlargement |  | Severe mitral stenosis |  | Left atrial enlargement |  |  |
|  | 41 | ST-T change due to ventricular hypertrophy |  | Hypertrophic cardiomyopathy | HR:59bpm | Asymmetric left ventricular hypertrophy; Systolic anterior motion of the mitral valve, obstructive form |  | 1 week ago |
|  | 42 | Right ventricular hypertrophy |  | Right heart failure  Pulmonary hypertension |  | Right heart enlargement;  Pulmonary hypertension |  | 2 days ago |
|  | 43 | ST-T change due to ventricular hypertrophy |  | Hypertension |  | Left ventricular hypertrophy | metoprolol |  |
|  | 44 | ST-T change due to ventricular hypertrophy |  | Aortic stenosis | HR:52bpm  QT: 460/428ms | Left heart enlargement;  Aortic stenosis;  Left ventricular hypertrophy; |  | 5 days ago |
| Others | 45 | Brugada syndrome |  | Ventricular tachycardia | HR:57bpm |  |  | 1 day ago |
|  | 46 | S_1_Q_3_T_3_ pattern |  | Acute pulmonary embolism | QRS: 151ms | TnI: 0.29ng/ml;  Pulmonary hypertension |  | 2 days ago |
|  | 47 | Epsilon wave |  | Arrhythmogenic right ventricular cardiomyopathy  Sustained ventricular tachycardia | HR: 54bpm  QRS: 140ms | TnI: 0.156ng/ml;  Regional right ventrivcular dyskinesia |  | 2 and 5 days ago |
|  | 48 | long QT interval |  | Non-ST-Segment elevation myocardial infarction  Paroxysmal atrial fibrillation | HR: 57bpm  QT:470ms | K: 1.9mmol/L |  | 1 and 2 days ago |
|  | 49 | long QT interval | Atrial fibrillation | Paroxysmal atrial fibrillation | QT: 464ms | Left atrial enlargement | metoprolol  amiodarone | 3 and 4 days ago |
|  | 50 | Takotsubo cardiomyopathy |  | Acute coronary syndrome  Heart failure | QT: 479ms | TnI:4.45ng/ml;  LVEF: 37%;  Apical ballooning |  | 2 and 3 days ago |

ECG,Electrocardiograph; HR, heart rate; LVEF, left ventricular ejection fraction; PR, PR duration; QRS, QRS duration; QT, QT interval; TnI, Troponin I;
